# A comprehensive review of COVID-19 symptoms and treatments in the setting of autoimmune diseases

**DOI:** 10.1186/s12985-023-01967-7

**Published:** 2023-01-07

**Authors:** Zahra Hamidi, Shaghaiegh Jabraeili-Siahroud, Yalda Taati-Alamdari, Parisa Shiri Aghbash, Ali Shamekh, Hossein Bannazadeh Baghi

**Affiliations:** 1 Student Research Committee, Tabriz University of Medical Sciences, Tabriz, Iran; 2 Immunology Research Center, Tabriz University of Medical Sciences, Tabriz, Iran; 3 Department of Virology, Faculty of Medicine, Tabriz University of Medical Sciences, Tabriz, Iran; 4 Infectious and Tropical Diseases Research Center, Tabriz University of Medical Sciences, P.O. Box 5165665931, Tabriz, Iran

**Keywords:** Autoimmune diseases, COVID-19, Immune system, SARS-CoV-2

## Abstract

After the first reporting of the index case of Severe Acute Respiratory Syndrome (SARS)-CoV-2-associated disease at the end of December 2019, the virus spread quickly throughout the world, prompting the WHO on 11 March 2020 to declare the disease a global pandemic. The coronavirus disease 2019 (COVID-19) pandemic, raises concerns for all people, mainly for susceptible population. People with pre-existing diseases, especially individuals with autoimmune disorders, are more at the risk of SARS-CoV-2 infection because of compromised immune system due to frequent use of immunosuppressive drugs and steroids. Patients with autoimmune diseases and their physicians have concerns about these patients’ healthcare, since they are at a higher risk for COVID-19 infection, may show severe complications of COVID-19, and may experience probable flares of their pre-existing disease. Even though there have been several studies discussing the relation between COVID-19 and various types of autoimmune diseases, it cannot be ascertained that all patients with autoimmune diseases experience more severe complications of COVID-19 and have more hospitalization or mortality rate. The situation depends on each patient’s condition, such as the type and the severity of the underlying autoimmune disease and the kind of treatment they receive. In the present review, we have discussed the effects of COVID-19 pandemic on patients with different autoimmune diseases and their relative concerns about their treatments. As a result, we have reviewed further considerations that should be taken into account for these patients during the pandemic or when they are infected with COVID-19.

## Background

Coronavirus disease 2019 (COVID-19), which is caused by a highly infectious respiratory virus, has imposed devastating effects throughout the world in recent years [1]. According to the World Health Organization (WHO), it is responsible for the death of almost 6 million people worldwide and has been known as the most significant global health crisis since 1918’s influenza epidemic [1]. The prominent issue in the management of COVID-19 is respiratory system damage. The virus may initiate cytokine storms, a condition in which extreme immune system responses and extensively activated immune cells may induce hyper inflammation [2–4]. COVID-19 infection also affects other organs; therefore extrapulmonary clinical symptoms can be observed in the cardiovascular, nervous, urinary, and reproductive systems [5, 6].

**Fig. 1:**
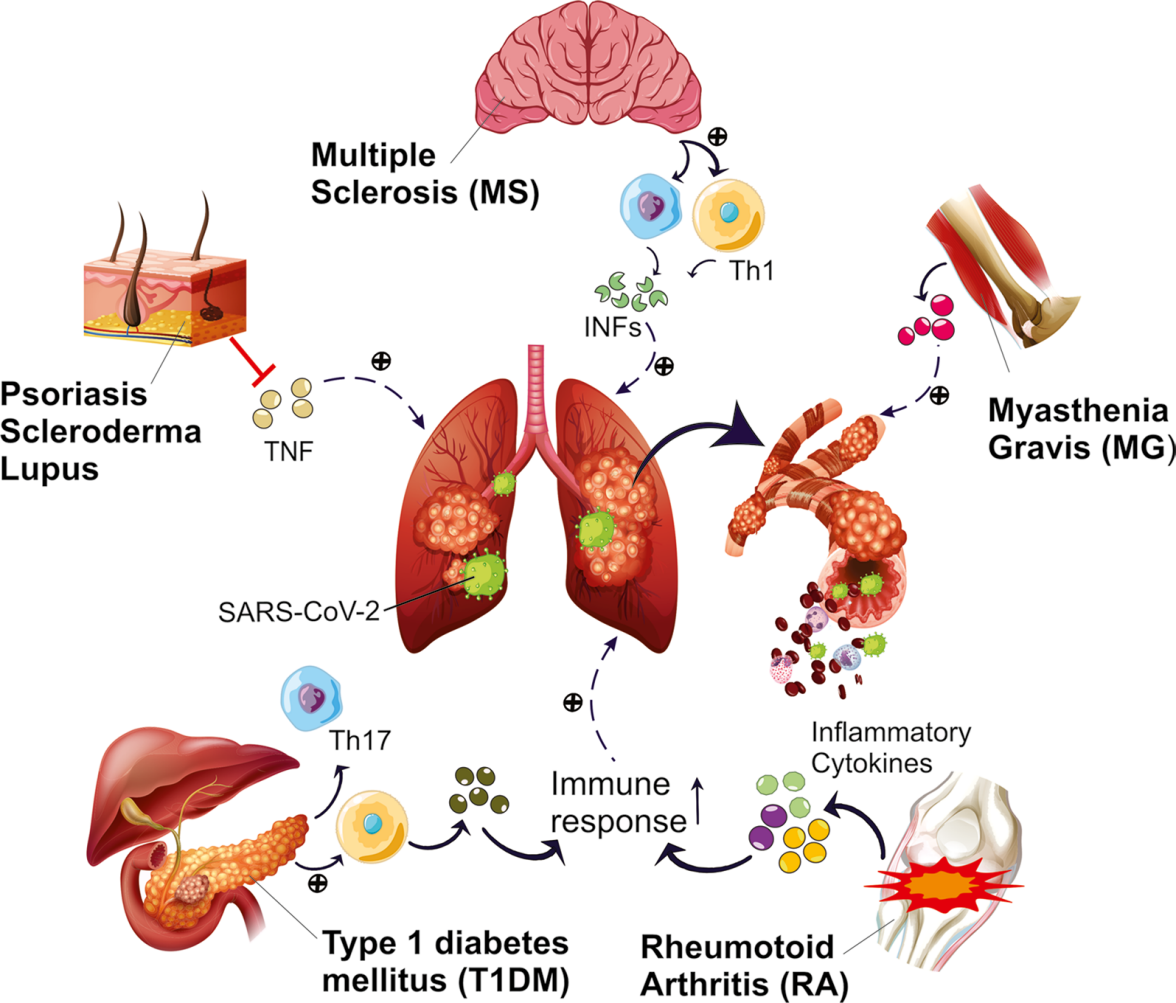
The effects of autoimmune diseases on the severity of COVID-19. Autoimmune diseases, due to the hyperactivity of immune cells’ function and the subsequent secretion of inflammatory and pro-inflammatory cytokines, lead to the increased expression of ACE-2, facilitating viremia. These events, thus exacerbate pulmonary disorders caused by SARS-CoV-2. *TNF* Tumor necrosis factor, *IFN* interferon

The coronavirus epidemic has severely affected health systems around the world. It has also caused a steep loss of livelihood due to its devastating influence on the worldwide economy and the mandatory limitation of jobs [1]. The propensity to the diagnosis and management of non-COVID-19 patients have decreased, and all the specialties have been affected by the reorganization of healthcare into hubs and spokes. On the other hand, telemedicine has proven to be beneficial to the described situation [7]. According to Yang et al. COVID-19 has different psychological effects on people, which can be more noticeable and prevalent within specific groups. These groups include survivors, patients with COVID-19 who have the fear of death, and the recovered patients who have the fear of rejection and distance from others [8]. Also, some groups are more at the risk of infection, including people over the age of 60 and people with pre-existing diseases [1]. Autoimmune Diseases (ADs) with inflammation, which are defined by the generation of autoantibodies and the provocation of inflammatory responses, are among the pre-existing diseases. As a result of impaired immune regulations and the loss of immune tolerance, ADs lead to the injury and dysfunction of target organs [9]. There have been increasing concerns about the patients with ADs during the COVID-19 pandemic. Furthermore, immunosuppressive medications, which are used in these patients, may also contribute to the increased risk of COVID-19 infection [10]. Several studies have hypothesized that these patients are at a higher risk of premature death and morbidity from infectious diseases; thus it can be assumed that SARS-CoV-2 infection would also cause severe complications in these individuals [11] (Fig. 1).

In this study, we tried to review the effects of COVID-19 and its global pandemic on patients with autoimmune diseases; and whether autoimmune disorders, as a pre-existing condition, can increase the risk of COVID-19 in patients with ADs or not. Furthermore, we have investigated the considerations that should be taken into account in the case of these patients, both during and after COVID-19 infection.

## Common autoimmune diseases

It is well known that some autoimmune diseases are more common and affect larger proportions of the population. In the following section, we have discussed some common autoimmune diseases for which sufficient data is available from the affected communities during the COVID-19 pandemic, as well as their respective considerations that should be taken into account.

### Type 1 diabetes mellitus (T1DM)

Type 1 diabetes mellitus is among the most common autoimmune diseases in infancy and childhood [12]. Previously, it was mostly considered a chronic disease which primarily revealed itself only in children and the adolescents; but in the past decade, it has been realized that age is not a restricting factor for the symptomatic onset of T1DM [13]. In this genetic-based autoimmune condition, CD4 + and CD8 + T cells have an increased auto-reactivity against B-cells [14], resulting in an impaired immune system function. In this regards, the pathogenesis of this disease consists of three stages: the formation of islet autoantibodies as stage 1; dysglycemia due to the destruction of beta cells in stage 2; and finally, the start of clinical symptoms in stage 3 [15].

Shi et al. in 2020 have indicated that diabetes and hyperglycemia (causing cytokine dysregulation) are serious risk factors for different bacterial and viral infections, including coronavirus infections and their complications, and mortality [16]. However, the existing data concerning T1DM are still scarce [14]. In recent cohort studies from Italy and China, the authors found no T1DM patients in the hospitalized cases of SARS-CoV-2, suggesting the possibility that the immunological characteristics of T1DM might provide some protection [14]. In other related studies, this low incidence of COVID-19 in T1DM individuals was considered to be due to several factors. Firstly, the overall younger age of T1DM patients makes them more likely to experience a milder COVID-19 and have a better prognosis. Also, in some countries, including Italy, T1DM individuals were considered as a high-risk group, and may have benefited from the early appliance of social distancing measures, resulting in decreased prevalence and less adverse outcomes. Indeed, it is possible that some patients with T1DM could have been infected with SARS-CoV-2 despite being asymptomatic [17]. Moreover, some studies reported that COVID-19 causes ketoacidosis and increases the risk of diabetic ketoacidosis (DKA) in patients with diabetes, which can be a reason for the increased length of hospitalization and mortality rate in these cases [18].

Furthermore, “lockdown” imposes several adverse effects on the individuals with T1DM, such as decreased physical activity, fewer interactions with peers, reduced healthy dietary intake, and psychological stress, which all can aggravate the disease condition [14]. Besides, parents of children with T1DM are at the risk of psychological distresses related to post-traumatic stress disorder (PTSD). Thus, psychological and mental symptoms should also be considered during the pandemic [12].

Diabetic patients should measure their blood glucose and ketone levels frequently to balance their glycemic index appropriately. Patients with poor glycemic control are at the risk of severe complications, long hospitalization, and higher overall mortality [14]. There were contradictory results according to the studies that examined the impact of lockdown on glycemic control among T1DM patients. Some studies have reported serious glycemic control imbalances during the pandemic lockdown [19]. On the other hand, in a study conducted by Nwosu et al., there were no remarkable changes reported in the glycemic control of US children during the 2020 lockdown [20]. Also, an Italian research has unexpectedly reported a significant improvement in the glycemic control of young T1DM patients during the lockdown as a potential result of the reduced daily stress and the adaptation of a healthier lifestyle [21].

The experiences from managing T1DM patients who were infected with SARS-CoV-2 demonstrate that hydroxychloroquine can decrease insulin degradation, and thus cause hypoglycemia [14]. In contrast, antiviral drugs such as ritonavir and lopinavir may worsen the glycemic control and cause hyperglycemia [22]. Glucocorticoids, which form an integral part of the COVID-19 hospitalized patients’ treatment regime, can also lead to significant hyperglycemia [14].

### Systemic lupus erythematosus (SLE)

Systemic lupus erythematosus is an autoimmune disease that causes damage to multiple organs and tissues through disrupting the immune system function and activation of immune cells against autoantigens [10]. Compared to organ-specific autoimmune diseases, SLE displays some signs and symptoms by affecting several organ systems [23]. Because of the increased predisposition to many infections, repeated flare-ups, the resulting accumulation of damage, and also many other comorbidities in the longer term, so mortality and morbidity rates may increase in SLE patients [23, 24].

Although patients with ADs -SLE within this group- have a higher prevalence of COVID-19 [25], there are some cohort studies which have shown no increase in SARS-CoV-2 risk of infection in these patients [26]. Studies reported an increased mortality rate in the patients with systemic autoimmune rheumatic diseases compared to the general population [27]; but this increase has not yet been determined whether it is solely due to COVID-19, or the delayed diagnosis and treatment of their pre-existing systemic autoimmune rheumatic diseases [28].

Since SARS-CoV-2 enters target cells by using angiotensin-converting enzyme 2 (ACE2) as a receptor [29, 30]; thus, the overexpression of the ACE2 gene in lupus patients facilitates viral entry and increases viremia [31]. Moreover, the involvement of the renal and cardiovascular systems in SLE patients, restricted ability to mount an efficient immune response against the virus, the common use of immunosuppressive drugs and B-cell depleting treatments, predispose them to higher risks of infection, more severe complications, and worse outcomes [24, 32].

The most common SARS-CoV-2 clinical manifestations in lupus patients are fever, anosmia and cough [26]. Patients with pre-existing lupus disease may have flares during the course of COVID-19, including worsening or different clinical signs, manifestations, and laboratory parameters indicating involvement of one or more organs [33, 34]. For instance a case of 62-year-old man with SLE disease, developed coombs positive hemolytic anemia and antiphospholipid antibody syndrome after infection with SARS-CoV-2 [34].

SLE patients are susceptible to developing psychiatric comorbidities, and have endured several challenges and concerns during the COVID-19 pandemic [35]. Even though the clinically extremely vulnerable group (CEV) were forewarned to ‘shield’ during the first UK lockdown, shielding seems to have been negatively affecting the mental health of SLE patients and those with underlying diseases [28].

Several immunomodulatory treatments such as Tocilizumab which usually administered in the case of SLE, might be valuable for the management of immune response against SARS-CoV-2 [36]. However according to a multicenter Italian cohort, the overall results are indecisive [26].

A point to consider regarding SLE patients is that their laboratory parameters should be monitored regularly [37]. However, a survey of 1517 autoimmune disease patients revealed that those who had missed doctor appointments and had not accessed telehealth services, often stopped taking their medications without consulting their physician [38].

### Multiple sclerosis (MS)

Multiple sclerosis is a chronic inflammatory, autoimmune neurodegenerative disease of the central nervous system resulting in the impairment of nerve conduction, and is associated with immune system malfunctioning [39]. The exact cause of MS has not yet been determined; however, researchers believe that a combination of environmental and genetic factors may cause its pathogenesis. The main event in the pathogenesis of this autoimmune disease is associated with the stimulation and activation of two subtypes of CD4 + cells, including Th1 and Th17, by an unknown antigen [40]. According to studies, human leukocyte antigen (HLA) is an essential genetic factor influencing the incidence of the disease. Environmental factors such as vitamin D deficiency in higher latitudes and various infections such as Epstein-Barr virus (EBV) may affect MS pathogenesis [40].

Patients with MS manifest vision, vestibular, urinary and bowel symptoms; cognitive, psychiatric and bulbar dysfunction; and also motor and sensory weaknesses [40]. They require long-term immunosuppressive medications or immunomodulating disease-modifying therapies (DMTs) [41]. A DMT is needed to assist in the control of MS patients’ course of disease, and decrease the risk of relapses [42]. The risk of infection increases as a result of the immune system dysfunction induced by the pre-existing autoimmune disorders and immunosuppressive medications. Even though immunosuppressive drugs have made a significant contribution to the control of MS progression; these drugs can also increase the risk of parasitic, fungal, bacterial, and viral infections in these patients [43]. Considering the spread of COVID-19 around the world, MS patients in the present situation could theoretically be considered at increased infection risks and its resulted health anxiety [43]. Moreover, the COVID-19 pandemic has also impacted the required healthcare for MS patients. Some patients stopped their DMTs without seeking medical advice, and others missed appointments, drug infusions, and refills [42]. A nationwide analysis from Germany, which included people with MS hospitalized from January 1st 2019 to December 31st 2020, revealed that during different pandemic phases, the hospitalization of patients with a first diagnosis of MS, relapsing-remitting MS, and primary and secondary progressive MS decreased markedly. The most substantial declines occurred in the patients with progressive MS at the first wave of COVID-19 pandemic [44]. This suggests that new MS cases might had been missed in this period of time.

Patients with MS and MS specialists are facing many challenges during the present COVID-19 pandemic:

1. Concerns regarding DMT treatment.
2. Logistical problems in dispensing DMT.
3. Insufficient hospital beds.
4. Lack of standard guidelines regarding maintenance therapy and relapse prevention in the setting of COVID-19.
5. Coordinating the efforts to raise awareness about the general and specific issues of infection control among the patients and medical community.
6. Concerns about the utilization of immunosuppressive or immunomodulatory treatments by the patients [45].

Although DMTs may increase the risk of infection in patients with MS, it has been highlighted that poorly controlled MS could impose more significant damages than COVID-19 [39].

COVID-19 can cause symptoms such as fever, cough, shortness of breath and fatigue respectively in both MS patients and general people. It is noteworthy that fatigue is the most common complaint in MS patients and that it worsens with COVID-19, as an infection. So it has been reported with 5.2% higher incidence in MS patients than in non-MS patients. A few asymptomatic MS patients have also been reported in the studies[46].

Studies in MS patients and the general population with confirmed COVID-19 include the following:

- Less than 50% of MS patients with coronavirus needed to be hospitalized.
- There was no difference in hospitalization rates between patients with MS and the general population in these studies; hospitalization occurs more frequently among older patients with a progressive condition and a greater level of disability. Additionally, more comorbidities and obesity were found among the hospitalized patients; these patients were mostly men [46].
- Among those admitted with MS, most hospitalized patients did not utilize DMTs, and later included the following: B-cell depletion therapies, teriflunomide, and fingolimod [46].
- Mortality rates were also reported more or less in patients with no DMTs, B-cell depletion, interferon therapy, teriflunomide, and natalizumab [46].
- There has been general agreement that interferon and glatiramer acetate therapy for MS patients does not increase COVID-19 risks, and interferon interventions might even be protective. Some studies declare that high-efficacy medications such as sphingosine-1-phosphate (S1P) receptor modulators, B-cell depleting therapies, alemtuzumab, and cladribine might be associated with increased COVID-19 susceptibility in patients with MS. Patients with high-efficacy treatments were less likely to develop COVID-19 than those without DMTs [46].

A summary of the mechanisms of action and recommendations for some DMTs used for MS patients during the coronavirus pandemic is shown in Table 1.

In general, COVID-19 does not seem to cause significant offend or mortality in patients with MS. However, further larger investigations in this topic are required to study this matter more closely while adjusting for COVID-19 risk factors. Overall, the use of DMTs does not appear to pose as a significant risk factor for poor COVID-19 outcomes; however, there is a possibility that the usage of B-cell depleting therapies could exacerbate SARS-CoV-2 infections [46].

### Rheumatoid arthritis (RA)

Rheumatoid Arthritis is an autoimmune disease characterized by the immune-mediated inflammation of synovial joints and extra-articular involvements. There are still no definite causes identified for this disease. As a result of untreated joint inflammation, cartilage and bone around the smaller peripheral joints gradually erode, and the disease can eventually spread to the proximal joints. In the early and established RA, the symptoms last shorter and longer than six months respectively [47]. RA has an unknown etiology; it is thought to be a result of both genetics and the environment [47, 48]. Patients with RA have a higher rate of functional impairment, affecting at least 15.8% of them. This resulted disability leads to decreased productivity, increased absenteeism, increased medical expenses, and decreased quality of life [49, 50]. As a result of the COVID-19 pandemic’s impact on the economy, individuals are at an increased risk of mental health disorders like depression and anxiety. Depression and suicide are both associated with the functional impairment seen in RA patients. Also the sensation of pain and tenderness may be more intense for those who display mental distress [49]. Based on a longitudinal observational study among patients with autoimmune inflammatory rheumatic diseases and COVID-19, 44% of the sample required hospitalization, especially the elderly who also had multiple comorbidities [51].

Studies have revealed that remote counseling is effective for quarantined RA patients. Telemedicine is an affordable method in the urban areas; but in rural areas, it is a challenge in terms of access to communicational equipment [49]. In addition, during the lockdown, the diagnosis of new disease cases becomes more difficult, and as a result, the initiation of treatment interventions is delayed [49]. People with RA are at higher risks of contracting an infectious disease compared to healthy individuals; since they utilize both synthetic and biological disease-modifying drugs. As a result, the COVID-19 outbreak should not prevent the patients from receiving RA treatments. Furthermore, stopping the ongoing therapies could lead to the need for corticosteroids (CS) as a bridging treatment, further increasing the risk of viral infection and misclassifying CS as an appropriate treatment for the interstitial pneumonia related to SARS-CoV-2 [52, 53]. Individuals with RA who take chloroquine or hydroxychloroquine as conventional synthetic disease-modifying anti-rheumatic drugs (csDMARDs), might benefit from this treatment strategy to prevent or reduce the severity of COVID-19 infection [52].

There is an increased mortality risk associated with glucocorticoids; while there may be an increased hospital admission risk with the use of methotrexate and rituximab. On the other hand, COVID-19 treatment may be affected by differences in drug mechanisms, emphasizing the importance of further investigating the immunosuppressive effects of the therapies. According to the related studies, in patients with rheumatic diseases, COVID-19 does not significantly affect mortality [54]. In comparison with the general population, chronic arthritis patients treated with disease-modifying anti-rheumatic drugs, both biologic and synthetic target medications, do not appear to be at a higher risk for respiratory severe conditions resulting from COVID-19 [55, 56].

### Vasculitides

Vasculitides are known as the inflammation of blood vessels and are categorized by the different kinds of vessels that the inflammation affects [57]. As studied in some research, it is anticipated to confront a higher rate of SARS-CoV-2 infections in vasculitis patients who had poor disease control [58]. SARS-CoV-2 testing rates are significantly higher in patients with Immune-Mediated Inflammatory Diseases (IMID) than in non-IMID patients, especially in those with vasculitis (3124.1 per 10,000 population) [59]. Patients with a rheumatic disease (such as vasculitis) had a higher hospitalization rate, and more frequently required intensive care or oxygen therapy [60]. However, other results showed that most of the patients with mild COVID-19 did not require ICU care or even hospitalization [58]. Studies indicated that none of these patients have used antiviral drugs to treat COVID-19 [58]. In this way, there were no increases observed in the hospitalization rates for COVID-19 in rheumatic vasculitis patients treated with anti-rheumatic drugs (e.g., methotrexate) alone or in combination with Janus kinase inhibitors [60, 61]. Hospitalization rates also did not rise in COVID-19 patients following treatment with non-steroidal anti-inflammatory drugs (NSAIDs) and hydroxychloroquine; but those treated with tumor necrosis factor (TNF) inhibitors had a decreased rate of hospitalization, and those treated with prednisone showed an increased rate [60]. Another research from Spain reported that among ten systemic vasculitis patients treated with rituximab, two cases died as a result of this treatment. These investigations found a correlation between rituximab and the negative outcomes for SARS-CoV-2 infection [62]. Among the commonly used treatments, 44.73% of the patients were treated with azithromycin and 15.78% with ivermectin [58]. Although immune suppressants weaken the immune system, some have been used for mild COVID-19 patients, such as NSAIDs, colchicine, dapsone, tocilizumab, low-dose methotrexate, and intravenous immune globulin (IVIG) [63].

### Psoriasis

Psoriasis is a chronic inflammatory skin disease, commonly presented with silvery-scaled plaques, which can also affect the scalp, eyes, and joints [64]. The medications for this disease are corticosteroids, immunosuppressants, or tumor necrosis factor-alpha counterparts (Anti-TNFα agents); similar to other immune-mediated inflammatory disorders. These medicines can reduce the immune responses in patients; thus, they affect the clinical course and outcome of COVID-19 [65]. In this regard, Karadag et al. reported that as the (Anti-TNFα) affect the upper respiratory tract, they can also increase the risk of SARS-CoV-2 infection [63]. Moreover, immune suppressive medications can mask the typical COVID-19 symptoms in the first stages, and therefore may hinder its diagnosis and make its treatment difficult [65]. Other studies have mentioned a few differences in COVID-19 symptoms in psoriasis patients and other diseases [66]. Most of the studies insisted that there is a higher risk of COVID-19 in psoriasis patients; but there is a low rate of mortality recorded for this group [59, 67]. The findings indicate that patients with autoimmune diseases who were hospitalized for SARS-CoV-2 infection had a lower need for staying in the intensive care unit (ICU) and mechanical ventilation (MV), and are not significantly more prone to severe disease [66]. It has been reported that one third of patients who discontinued their immunosuppressive therapy before [68] or during the pandemic had developed SARS-CoV-2 infection [66].

Treatment of autoimmune diseases is one of the most important subjects discussed in the setting of COVID-19. In this regard, one study about immunosuppressive therapy in COVID-19 patients with psoriasis reported that the patients receiving Apremilast show a low risk for infection. They also noted that Apremilast doesn’t increase the risk of pulmonary fibrosis, one of the common causes of COVID-19 mortality in psoriasis patients [69].

## Rare autoimmune diseases

Given to the previous sections and the discussion about common autoimmune diseases, here we describe two relatively rare autoimmune diseases that deserve more consideration during the SARS-CoV-2 outbreak: Myasthenia Gravis and Scleroderma.

### Myasthenia gravis (MG)

Myasthenia gravis is a chronic autoimmune neuromuscular disorder [70], and is characterized by spasmodic muscle weaknesses usually affecting the bulbar, ocular, respiratory, and limb muscles [71]. The condition is believed to be caused by the autoantibodies that block the neuromuscular transmission [72]. The risk of contracting viruses such as SARS-CoV-2 may be increase in MG patients for several reasons, including immunosuppressive therapies [73] and the diaphragm muscle weakness which can lead to respiratory complications [74]. Infectious conditions can exacerbate MG and lead to a myasthenic crisis, characterized by a restrictive respiratory failure. Essential and successful treatments during a myasthenic crisis include intravenous immunoglobulin (IVIG) therapy and plasmapheresis (PLEX) [75].

The decision regarding whether to continue immunosuppression or initiate acute interventions, like high-dose corticosteroids or IVIG, in MG individuals affected with COVID-19 should be made case-by-case regarding each patient’s condition and the severity of both COVID-19 and MG. Since COVID-19 manifests a mild to severe spectrum of symptoms, and also the majority of MG patients manifest the milder form, the standard treatment for MG should either be continued or may require an increase in the dosage of corticosteroids. However, in severe cases requiring hospitalization, immunosuppression may need to be temporarily paused, especially in the presence of concurrent infections or the occurrence of sepsis. It is best to avoid immune depleting agents, while more stable immunosuppressive drugs such as azathioprine and mycophenolate, can be continued [76].

Nevertheless, increasing evidence suggest that immunosuppression might have protective effects through immune response limitation, which in turn might cause an inflammatory cytokine cascade deteriorating the clinical condition of patients [77, 78]. Another cohort study declares the unlikeliness of the negative impact of immunosuppression therapy on this group, suggesting that these therapies can be continued [79]. However, individualized treatment decisions must be made according to every patient’s general health status and any likely comorbidities [75].

In an observational study on 15 MG adult patients admitted with COVID-19 has shown that most COVID-19 hospitalized patients with previous MG had a more severe disease course compared to otherwise non-MG patients [73]. The use of PLEX therapy and IVIG have demonstrated favorable outcomes regarding the treatment of patients with MG exacerbation [80]. Accordingly, early immunotherapy (PLEX and IVIG) and neurological consultations are recommended for MG patients with severe COVID-19 conditions [73].

Some reports have illustrated that the usage of prednisone and a second immunosuppressant did not lead to additional unfavorable effects in the MG cases [70, 81]. There is significant evidence to suggest that MG patients with COVID-19 development should avoid hydroxychloroquine and azithromycin because of precipitation of the crisis [82]. Tocilizumab has been shown to be effective in a patient with a history of myasthenic crisis who presented with COVID-19 [70]. It should be noted that according to the previously discussed study, the continuous use of neuromuscular blocking agents (NMBs) for most of the ventilated patients, led either to their deaths or their prolonged hospitalization, suggesting the necessity for the cautious implementation of these agents alongside antibiotics [73].

### Scleroderma

Scleroderma is another rare autoimmune disease involving the connective tissue [83], characterized by the skin’s hardening and thickening [84].

As most of the systemic sclerosis (SSc) patients have interstitial lung diseases and take immunosuppressive drugs as a major part of their treatment, we need to categorize them in a high risk group for COVID-19 [85]. They are also at a higher risk for mortality in the COVID-19 pandemic [27]. In systemic sclerosis, endothelial injury causes vascular leakage, promoting inflammation in the lungs, heart, and other organs [86]. Such pre-existing lung fibrosis may deteriorate COVID-19 infection in SSc patients [87]. There is substantial evidence that the endothelium plays a significant role in the pathogenesis of both SSc and COVID-19 [88].

P. Brito-Zerón et al. studied on four scleroderma patients who received rituximab and one who used tocilizumab. The three patients receiving rituximab, required ICU and one dead. However, the patient who used tocilizumab had a milder course of COVID-19 despite insulin-induced T2DM [32].

**Table 1:** Disease-modifying therapies (DMTs) recommendations for patients with Multiple Sclerosis (PwMS) during COVID-19 pandemic

Type of DMT	The mechanism of action	Indication of DMT initiation and maintenance during COVID-19 pandemic	References
Interferon-Beta (INF-β)	T-cell proliferation and leukocyte migration across the blood-brain barrier (BBB) are inhibited by the down-regulation of MHC expression on the APCs and the suppression of pro-inflammatory cytokines	Can be implemented. If influenza-like symptoms reoccur, it should be discontinued until a definite diagnosis can be made	[45, 89]
Glatiramer Acetate	Transforms inflammatory T-helper-1 cells into regulatory T-helper-2 cells	Can be implemented. Systemic risk of infection is not increased when using this medication	[45, 89]
Teriflunomide	Inhibition of cytostatic effects by reversible and selective targeting of T and B cells	Can be implemented. Should be discontinued in COVID-19 confirmed cases	[45]
Dimethyl Furmarate (DMF)	Reduction of pro-inflammatory cytokines and lymphocyte entry into the CNS	Can be implemented. Should be discontinued in COVID-19 confirmed cases	[45]
Fingolimid	Isolation of lymphocytes in secondary lymphoid tissues	Not indicated. It is associated to an increased risk of infection	[45, 90]
Natalizumab (NTZ)	Compromising immune supervision only in the CNS by preventing the active lymphocyte trafficking through the blood-brain barrier	It can be initiated and maintained if patients are not infected by COVID-19. Should be discontinued in COVID-19 confirmed patients	[45]
Cladribine	Optional reduction of CD19 + B lymphocytes and T lymphocytes	Not indicated	[45, 91, 92]
Rituximab	Cellular cytolysis of CD20-expressing B lymphocytes via a selective connection to these cells, and activating complement mediated cell lysis	Not indicated	[45, 91, 92]
Ocrelizumab	Cellular cytolysis of CD20-expressing B lymphocytes via selectively connecting to these cells, and the activation of complement mediated cell lysis	Not indicated	[45]
Alemtuzumab (AMZ)	Induction of T and B cell depletion through antibody-dependent cellular cytotoxicity	Not indicated	[45]

*APC* antigen presenting cell, *MHC* major histocompatibility complex

## Conclusion

Concerns about the patients with ADs, both common and rare, have increased with the outbreak of the COVID-19 and the lockdown situation. COVID-19 induces greater anxiety among patients with ADs compared to healthy individuals, partly due to the use of immunosuppressives, the impaired physiological function of the organs as a result of autoantibodies, and the reduced attention and poor healthcare of this group of patients during the epidemic. Furthermore, the psychological burden of lockdown has a significant effect on the overall health condition of these patients. The situation of patients with both ADs and COVID-19 infection depends on their treatment and care; each of the antiviral and immunomodulatory therapies or drugs used by these patients imposes different effects, such as worsening or improving their condition. According to research, immunomodulatory treatments have had an important impact in reducing the severity of ADs. However, it is challenging to determine with confidence whether having an autoimmune illness affects the mortality rate of SARS-CoV-2 and the severity COVID-19 or not, especially when a dilemma is present between addressing the existing disease or the new infection.

## Data Availability

The data that support the findings of this study are available from the corresponding author upon reasonable request.
